# Occupational Exposure to Antineoplastic Drugs in Twelve French Health Care Setting: Biological Monitoring and Surface Contamination

**DOI:** 10.3390/ijerph20064952

**Published:** 2023-03-11

**Authors:** Sophie Ndaw, Aurélie Remy

**Affiliations:** Department of Toxicology and Biomonitoring, French Research and Safety Institute for the Prevention of Occupational Accidents and Diseases (INRS), F-54519 Vandoeuvre les Nancy, France

**Keywords:** antineoplastic drugs, cytotoxic drugs, exposure assessment, human biomonitoring, healthcare workers, occupational exposure, urine, environmental monitoring

## Abstract

Antineoplastic drugs used in the treatment of cancers have an intrinsic toxicity, because of their genotoxic, teratogenic, and carcinogenic properties. Their use is recognized as an occupational hazard for healthcare workers (HCWs) who may be exposed. The purpose of this article is to present biological- and environmental-monitoring data collected in twelve French hospitals over eight years. Urine samples were collected from a wide range of HCWs (250 participants) from pharmacy and oncology units, including physicians, pharmacists, pharmacy technicians, nurses, auxiliary nurses, and cleaners. The investigated drugs were cyclophosphamide, ifosfamide, methotrexate, and α-fluoro-β-alanine, the main urinary metabolite of 5-fluorouracil. Wipe samples were collected from various locations in pharmacy and oncology units. More than 50% of participants, from all exposure groups, were contaminated with either drug, depending on the unit, the day, or the task performed. However, workers from oncology units were more frequently exposed than workers from pharmacy units. Significant contamination was detected on various surfaces in pharmacy and oncology units, highlighting potential sources of exposure. Risk-management measures should be implemented to reduce and maintain exposures at lowest-possible levels. In addition, regular exposure assessment, including biological and environmental monitoring, is recommended to ensure the long-term efficiency of the prevention measures.

## 1. Introduction

Antineoplastic drugs (ANDs), also known as cytotoxic drugs, are widely used in cancer therapy because they can inhibit cancer growth by disrupting cell division and killing actively-growing cells. Most of these agents have genotoxic, reprotoxic, and/or carcinogenic properties [1]. As healthcare workers (HCWs) may be exposed to cytotoxic drugs, the use of such drugs has been recognized since the late 1970s as an occupational hazard and has therefore become a major concern for occupational medicine in hospitals. The National Institute for Occupational Safety and Health (NIOSH) alert was published in 2004 [2] to increase HCWs’ awareness of the health risks posed by working with hazardous drugs and to provide them with measures for protecting their health. Occupational exposure to ANDs can occur at all stages of their use, including preparation, administration, and waste disposal. Contamination results primarily from transcutaneous penetration [3] or inhalation of aerosols during the handling of ANDs or patient excreta [4]. Several others prevention guides have been issued since the NIOSH alerts, setting out recommendations regarding how to limit exposure and to guarantee the safety of healthcare workers [5,6]. The guidelines are directed towards professionals likely to be in direct contact with these drugs or with patient excreta, specifically nurses, pharmacists, physicians, pharmacy technicians, and auxiliary nurses. In 2016, the European Parliament published a document featuring 11 recommendations, that urged the European Commission to take the necessary measures to promote prevention practices and implement legislation aimed at protecting health professionals handling cytotoxic drugs [7].

However, studies published since the 2000s continue to report contamination of HCWs and frequent contamination of hospital work surfaces [8,9,10]. Biological monitoring has been used for a number of years to demonstrate the contamination of healthcare workers by ANDs. As a result, biological monitoring also accounts for the effectiveness of the risk-management measures implemented. The most studied drugs have been cyclophosphamide, ifosfamide, methotrexate, platinum salts, and 5-fluorouracil [8,11,12,13]. For its part, the determination of environmental contamination is a useful tool for assessing the effectiveness of cleaning procedures and increasing the awareness of HCWs about the main sources of contamination [14,15].

In this paper, we present the exposure data collected in 12 French hospital centers over eight years. The urine concentrations of cyclophosphamide (CP), ifosfamide (IF), methotrexate (MTX), and α-fluoro-β-alanine (FBAL), which is the main urine metabolite of 5-fluorouracil (5FU), were measured for a population of physicians, pharmacists, pharmacy technicians, nurses, auxiliary nurses, and cleaners. To better understand factors that affect exposure, measurements of surface contamination, using cyclophosphamide and 5-fluorouracil as markers, were also performed in pharmacy and oncology units.

## 2. Materials and Methods

### 2.1. Study Design

Twelve medical centers, including four cancer-treatment centers, six general hospitals, and two clinics, took part in the study between 2008 and 2016. ANDs were prepared at a central drug-compounding unit within the hospital pharmacies, except for two centers. In those two hospitals, drug-compounding was performed by nurses in the oncology ward. Biological safety cabinets or isolators, located in dedicated rooms, were used for the preparation of ANDs. Trained pharmacy technicians were in charge of drug-compounding, under the supervision of a pharmacist. ANDs were then distributed in day-care oncology units or conventional oncology units for administration to patients.

Healthcare workers, including physicians, pharmacists, pharmacy technicians, nurses, auxiliary nurses, and cleaners, were recruited within hospital pharmacies and oncology units to take part to the exposure assessment. The participants were recruited on a voluntary basis. Workers who had a close family member undergoing chemotherapy were excluded from the study. The participants were provided with all research-related information and their written informed consent was received in advance. The study protocol was approved by the regional ethical committee CPP-EST III.

### 2.2. Collection of Contextual Information

A questionnaire designed to provide information about work activity was completed for each participant. Answers to the questionnaire specifically detailed the participants’ job, specific work tasks, ANDs handled, manipulation of excreta, personal protective equipment, possible incidents (bottle breakages, accidental spills, splashing, etc.).

### 2.3. Urine and Wipe Samples Collection

Urine samples were collected before the start and at the end of work shifts during a working week, for five consecutive days, from Monday to Friday. However, the working rhythm was usually different between hospitals and between units. A working rhythm based on daytime hours (from 7 a.m. to 7 p.m.) was the most common in pharmacy units, while rotating shifts (day shift or night shift) was regular in oncology units. Therefore, HCWs were monitored for 2 to 5 days during a working week, depending on their working rhythm, and they provided 4 to 10 urine samples. Urine samples were stored at −20 °C before analysis.

Wipe sampling was performed by a trained researcher with wipes impregnated with water (GhostWipe, Environmental Express) using a validated in-house method. The wipe taken from a flat surface was sampled in an area of 10 cm × 10 cm and in two directions, and then placed into a clean container. Other surfaces (gloves and door handles) were totally wiped by the researcher. Different locations likely to be contaminated by ANDs were sampled. The surfaces included infusion bags, ANDs vials, gloves, benches, door handles, computer mice, medical trays, etc.

### 2.4. Analytical Methods

Analysis of urine samples was performed via high-performance liquid-chromatography-tandem mass spectrometry, using a Varian 1200L Triple-quadrupole mass spectrometer system with an ESI interface (Varian, Les Ulis, France). The liquid chromatography system consisted of two Varian Prostar 210 pumps and a Prostar 410 autosampler.

For the quantification of cyclophosphamide and ifosfamide, 60 µL of internal standard (2 mg/L D6 deutered cyclophosphamide) were added to 6 mL urine sample. The mixture was vortexed and 5 mL were loaded on a Chromabond^®^ XTR cartridge, filled with kieselguhr phase for liquid-liquid extraction (Macherey—Nagel, Hoerdt, France). The analytes were eluted with 20 mL of tert-butyl ethyl ether, and the eluate was evaporated to dryness under a stream of nitrogen. The residue was dissolved in 500 µL of a mixture of 0.5% acetic acid/methanol (50:50) then injected into HPLC-MS/MS. The analytical column was a Discovery HS F5 (3 µm, 150 × 2.1 mm) from Supelco (Saint-Quentin Fallavier, France), and the mobile phase used a gradient of water and methanol. The flow rate was 0.2 mL/min, the injection volume 20 µL, and the column was thermostated at 30 °C. The triple-quadrupole mass spectrometer operated in positive mode and the precursor ions/product ions were *m*/*z* 261/140 for cyclophosphamide, 261/92 for ifosfamide, and 267/140 for the internal standard. The limit of quantification was 0.05 µg/L for cyclophosphamide and 0.1 µg/L for ifosfamide.

For the quantification of methotrexate, 60 µL of formic acid and 60 µL of internal standard (Aminopterin 2 mg/L, supplied by Sigma-Aldrich, Saint-Quentin Fallavier, France) were added to a 6 mL urine sample. The mixture was vortexed and 5 mL were loaded on a 500 mg/3 mL Isolute HAX SPE cartridge (Biotage, Uppsala, Sweden) conditioned with 2 mL of methanol and 2 mL of 1% formic acid. The cartridge was washed with 4 mL of 100 mM ammonium acetate and 4 mL of methanol. The analytes were eluted with 2 mL of a mixture of formic acid/methanol (2/98) and the eluate was evaporated to dryness under a stream of nitrogen. The residue was dissolved in 500 µL of 25 mM formic acid then injected into HPLC-MS/MS. The analytical column was a Discovery HS F5 (3 µm, 150 × 2.1 mm) from Supelco (Saint-Quentin Fallavier, France) and the mobile phase used a gradient of 25 mM formic acid and acetonitrile. The flow rate was 0.2 mL/min, the injection volume 20 µL, and the column was thermostated at 50 °C. The mass spectrometer operated in positive mode and the precursor ions/product ions were *m*/*z* 455/308 for methotrexate and 441/294 for the internal standard. The limit of quantification was 0.1 µg/L.

The urine samples were analyzed for FBAL using the method described by [8]. Briefly, this procedure is characterized by a precolumn FBAL derivatization with 2,4-dinitrofluorobenzene, followed by solid phase extraction sample cleanup. The chromatographic separation was achieved by hydrophilic interaction chromatography (HILIC) on a Sequant ZIC HILIC column (AIT, Houilles, France), and the quantification was performed by tandem mass spectrometry in negative mode. The limit of quantification was 1.0 µg/L.

Cyclophosphamide and 5-fluorouracil were quantified in wipe samples using an in-house validated method. These compounds were extracted from wipe samples via an ultrasonic bath of 30 min, including 14 mL of a mixture of ethyl acetate/isopropanol (85/15). The extract was evaporated to dryness under a stream of nitrogen. The residue was dissolved in 400 µL of 20 mM ammonium acetate before analysis on the HPLC-MS/MS system. The limits of quantification were 0.5 ng for 5-fluorouracil and 20 pg for cyclophosphamide. Wipe samples, in which cyclophosphamide or 5-fluorouracil were quantified, i.e., identified in measurable quantities, were considered as positive samples.

### 2.5. Statistical Analyses

Urine samples in which at least one of the four biomarkers was detected were considered as positive samples. Similarly, the participants were considered to be contaminated by ANDs when at least one of their urine samples was positive. Given the low number of positive urine samples, exposure was then defined using a binary variable, with the following modalities: ANDs absence or ANDs presence. The chi-squared independence test was performed to determine whether there was a significant association between the exposure variable and the categorical explanatory variables (number of preparation, and exposure groups). When the chi-squared independence test was statistically significant, a logistic regression model (with a random effect ‘worker’) was applied to model the relationship between the exposure variable and the explanatory variable. The logistic regression allows us to estimate the probability of exposure-presence based on the explanatory variable, expressed by an Odds Ratio (OR). Statistical analyses were conducted using Stata 12.1 software (https://www.stata.com/). The statistical significance threshold was set at 5%.

## 3. Results

### 3.1. Study Population

A total of 250 HCWs volunteered to participate in this study. They were 16 pharmacists, 56 pharmacy technicians, 104 nurses, 48 auxiliary nurses, 14 cleaners, 4 physicians, and 8 participants with miscellaneous jobs.

In the pharmacy units, personal protective equipment (PPE) specifically comprised work clothes (pants and jacket) and possibly a disposable protective gown, a mobcap, a medical mask, specific shoes and possible overshoes, a pair of triple (latex, neoprene, nitrile) gloves or a pair of double (latex and/or nitrile) gloves for drug-compounding, using an isolator or a biological safety cabinet, respectively. Workers not directly involved in ANDs preparation usually wore a pair of single (latex or nitrile) gloves and, more rarely, a pair of double gloves. The main activities of pharmacy technicians included the receipt of ANDs from suppliers, the preparation of medical trays, and ANDs compounding. The pharmacy technicians were also responsible for cleaning the drug-compounding unit work surfaces. The pharmacists were responsible for validating the manufacturing records and preparations, and for managing the pharmacy unit. In the pharmacy units, the investigated substances, CP, IF, MTX, and 5FU were handled on a daily basis.

In the oncology units, HCWs wore work clothes and possibly a gown, a medical mask, and a pair of single latex, vinyl, or nitrile gloves. Nurses were responsible for ANDs administration to the patients, except for the intrathecal methotrexate injections which were performed by physicians. Auxiliary nurses, in charge of patient care and cleaners, were responsible for room hygiene. In the oncology units included in this study, the investigated substances (CP, IF, MTX, 5FU) were not actively handled at the same time. ANDs handled in these units were highly dependent on the unit’s oncological specialty. While all four markers were usually handled daily in a day-care unit, CP and IF were the drugs mainly used in hematology-oncology units.

### 3.2. Biological Monitoring of Healthcare Workers Exposure

#### 3.2.1. By Medical Centers

The annual number of drug preparations varied between 3000 and 28,000, depending on the hospital (Table 1). Five hospitals out of twelve performed less than 20,000 AND preparations per year. Exposure was defined according to a binary variable, with the following modalities: absence or presence of biomarkers of ANDs in urine samples. The chemical nature of the ANDs measured in urine samples was not taken into account in defining exposure. Quantifiable concentrations of ANDs were found in 15% of all the urine samples collected (250/1703). Contamination of urine samples was not observed for the three participants from center 12. For the remaining eleven medical centers, the percentage of positive samples varied between 1% and 30% (Table 1).

The overall percentage of HCWs with at least one positive urine sample was 53% (132/250 participants). Except for center 12, center 2 had the lowest percentage of HCWs with positive samples, 5%. This percentage varied between 42% and 80% at the other ten centers (Table 1). At centers 5 and 8, which had no centralized drug preparation unit (Table 1), exposures were high considering the relatively small number of preparations performed annually (7500 and 8500, respectively). However, these high exposures could not be attributed solely to the absence of a centralized drug preparation unit, since the nurses performed both drug preparations and administration activities in the same day.

The contamination of HCWs generally appears to be sporadic rather than systematic, given the overall low number of positive urine samples. Most of the contaminated participants had one to three positive samples during the sample-collection week. However, around ten employees per work shift turned out to be systematically contaminated. 

All investigated substances, cyclophosphamide, ifosfamide, methotrexate, and FBAL, were quantified in the urine samples (Table 2). Nearly 85% of positive urine samples were collected at the end of the work shifts. The urine samples were generally positive for at least one biomarker. However, up to three ANDs biomarkers could be quantified in some samples. Cyclophosphamide and 5-fluorouracil metabolite FBAL were the most frequently detected in the urine samples. They were quantified in 9 and 10 medical centers, respectively. This high frequency of quantification was expected because these two drugs were among the most commonly used ANDs in medical centers. Concentrations ranged between 0.05 and 0.99 µg·L^−1^ for cyclophosphamide, between 0.10 and 0.44 µg·L^−1^ for ifosfamide, between 0.10 and 3.17 µg·L^−1^ for methotrexate, and between 1.0 and 24.5 µg·L^−1^ for FBAL (Table 2). The exposure levels reached up to 20 to 30 times the respective quantification limits.

#### 3.2.2. By Exposure Groups

Contamination of urine samples was found in all exposure groups, except for physicians (Table 3).

Pharmacists and pharmacy technicians had equivalent percentages of positive urine samples, 10% and 11%, respectively. The percentage of workers with quantifiable samples was also identical (approximately 44%) for these two groups. The pharmacists included in this study were not involved in ANDs compounding. One of their main activities was validation of the preparations performed by pharmacy technicians, before the preparations were dispensed to the oncological wards, which involved a constant handling of infusion bags. With regard to the pharmacy technicians, the questionnaire revealed that they were more likely to be contaminated when they were not directly involved in ANDs compounding. Tasks performed before and after the compounding step, such as reception of drug vials, preparation of trays, ANDs disposal, or unit cleaning, were the main sources of exposure.

In the oncology units, HCWs, who were in constant contact with patients under chemotherapy, were also contaminated. More than 50% of nurses and auxiliary nurses were exposed during the monitoring period (Table 3).

Analysis of the questionnaire did not reveal a systematic relationship between ANDs administration by nurses and their exposure. Nurses were contaminated even if they did not directly handle ANDs. Moreover, nurses reported that they also handled patient excreta, as did auxiliary nurses, who ensured patient hygiene and handled bedding. Most of the ANDs can be found unchanged in urines, feces, sweat, vomit, and saliva from patients, which were therefore significant exposure sources for HCWs. Questionnaire analysis also revealed irregular wearing of gloves by nurses, especially when handling infusion bags.

Cleaners were not in direct contact with ANDs or with patients. Nevertheless, cleaners were exposed as much as other groups, with 50% of them contaminated and 15% of their urine samples positive for ANDs. The questionnaires did not allow an accurate identification of the tasks responsible for the cleaners’ exposure.

Nevertheless, it was observed that auxiliary nurses and cleaners usually wore vinyl gloves that were short at the wrist and did not protect the forearms, especially when handling excreta or bedding, or when cleaning rooms. In addition, the wearing of gloves was found to be inconsistent for these workers.

Participants who did not work in oncology units, or who were not known to be in direct contact with ANDs, were similarly exposed (Table 3). This was particularly the case for couriers, included in the “Others” exposure group, whose main activity was to convey preparations in closed containers among different units.

#### 3.2.3. Influencing Parameters for HCWs Exposure

The association between the exposure of pharmacy technicians or pharmacists and the number of annual preparations was investigated. No significant association was found among these variables.

Moreover, the chi-squared independence test did not reveal a significant association between exposure and exposure groups. However, when grouping together, on the one hand, the exposure data from the pharmacy unit participants (pharmacists and pharmacy technicians) and, on the other hand, the exposure data from the oncology unit workers (nurses, auxiliary nurses, and cleaners), a significant association was found between these two groups and their exposure (*p* = 0.001). The exposure was more important for oncology unit workers than for pharmacy-unit workers. A logistic regression (with a ‘worker’ random effect) was then performed to model the unit-based exposure (pharmacy vs. oncology). The unit turned out to be a statistically significant parameter, with an odds ratio (OR) of 1.87 [95% confidence interval (1.16–2.93)], indicating that there was an 87% higher exposure risk for oncology-unit workers than for pharmacy workers.

### 3.3. Environmenal Contamination

**Figure 1:**
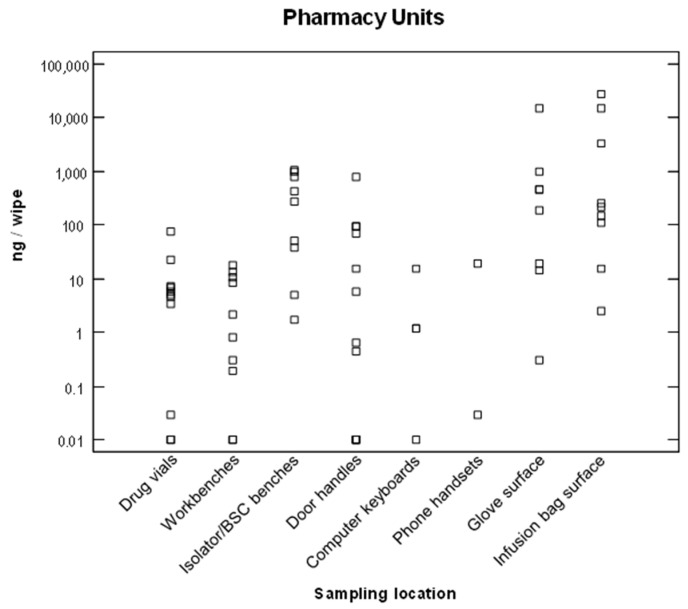
Surface contamination by cyclophosphamide or 5-fluorouracil (logarithmic scale) in pharmacy units (*n* = 63).

**Figure 2:**
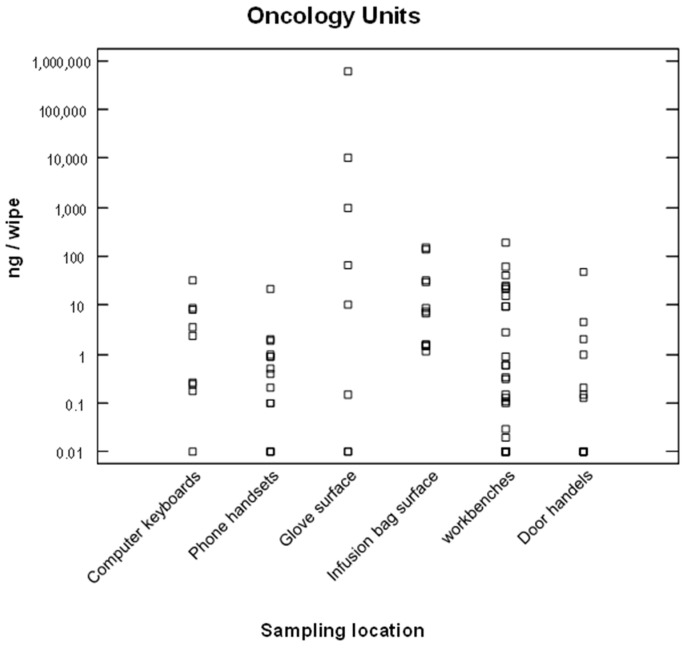
Surface contamination by cyclophosphamide or 5-fluorouracil (logarithmic scale) in oncology units (*n* = 84).

Environmental contamination by ANDs was measured at seven medical centers. Surface contaminations by 5-fluorouracil or cyclophosphamide in pharmacy and oncology units are represented in Figure 1 and Figure 2, on a logarithmic scale. The contamination levels were highly variable from one surface to another, or from one hospital to another, ranging from a 10 ng/wipe to 100 µg/wipe.

#### 3.3.1. Environmental Contamination in Pharmacy Units

Contaminations were regularly detected in pharmacy units on the isolator- or biological-safety cabinet workbenches, and on the surfaces of the pharmacy technicians’ gloves (Figure 1). High contamination levels up to 27 µg of ANDs were also measured on the surfaces of infusion bags. In the immediate environment of the isolators (or biological safety cabinets), other surfaces were also contaminated including the surface of drug vials, workbenches, door handles, telephone handsets, etc. These contaminations probably originated from the pharmacy-technicians’ gloves or from the chemotherapy preparations. At least 85% (54/63 samples) of the wipe samples collected in pharmacy units were contaminated with cyclophosphamide or 5-fluorouracil.

#### 3.3.2. Environmental Contamination in Oncology Units

Contaminations were also regularly detected in oncology units, mainly in the nurses’ rooms. Contaminated surfaces included the workbenches, the surfaces of infusion bags (which were often handled without gloves), and the surfaces of nurses’ gloves after hooking up or removing chemotherapy infusion bags. For example, a quantity of 572 µg of 5-fluorouracil was measured on a glove after a nurse had hooked up an infusion bag. Disposal of these contaminated gloves therefore represents a critical step regarding exposure.

Contaminations were also detected on door handles, computer keyboards, and phone handsets (Figure 2). About 73% (63/85 samples) of the wipe samples collected in oncology units were contaminated by cyclophosphamide or 5-fluorouracil. In addition, other surfaces (data not shown) such as floors, containers (or iceboxes) for infusion bags, medical trolleys, or different surfaces in patient rooms were also regularly contaminated.

## 4. Discussion

The aim of this study was to measure urinary ANDs levels in HCWs in order to highlight occupational exposure to chemotherapy drugs. A second objective was to identify the sources of exposure and to understand factors related to the tasks performed by HCWs that may affect workers’ exposure. Quantifiable concentrations of ANDs were found in some of the urine samples collected in our study, demonstrating that HCWs may be contaminated when handling chemotherapy drugs.

In the last two decades, several biomonitoring studies have been conducted to assess the occupational exposure to ANDS using urine-samples analysis. The data reported in the literature involves two to eleven medical centers, and the proportions of contaminated professionals were highly variable. A low percentage of contaminated workers, less than 20%, were reported in Europe [16,17,18,19,20,21]. Some other studies reported higher proportions of contaminated HCWs, >50% [22,23,24]. The results of these different studies are highly variable, probably due to the differences in study protocols, the investigated substances, or the limits of quantification of analytical methods. In addition, most of the available data relate to pharmacists, pharmacy technicians, and nurses. A systematic review of ANDs biological monitoring data was performed recently by [11].

In our study, the main steps of the lifecycle of ANDs at hospitals — including receipt, compounding, transport, administration, and disposal, but also the care of patients — were taken into account. The wide range of healthcare professionals involved in these tasks was included, in 12 medical centers. Therefore, this study provided exposure data not only on pharmacists, pharmacy technicians, and nurses, but also on physicians, auxiliary nurses, cleaners, and other miscellaneous workers. Four ANDs were selected as markers of exposure for this study, while more than thirty of them are commonly used in healthcare departments. The choice was driven mainly by the frequency and amount handled. In addition, CP, IF, MTX, and 5FU were among the most studied ANDs for exposure assessment [11]. However, these markers were not systematically handled at the same time by all participants. HCWs were exposed to either drug, depending on the unit, the day, or the task performed. Therefore, exposure was defined in this study with a binary variable: absence or presence of ANDs metabolites in urine samples.

More than 50% of study participants had quantifiable levels of the investigated substances in their urine. However, the exposure may be considered quite low and sporadic, as there was a high number of negative samples (ANDs were not detected in 85% of urine samples). Workers usually excreted ANDs in their urine one to three times during the week of the study, except for about ten employees who were found to be systematically exposed at each work shift.

The contaminations measured in this study were contrasting, depending on the hospital center. This was probably due to several factors including work organization (centralized or non-centralized drug-compounding unit), preventive measures, and workers’ observance or awareness of risk associated with handling ANDs, etc. While actual ANDs compounding appears to be a well-controlled task, because of a centralized preparation unit, use of an isolator or biological safety cabinet, wearing of pairs of triple or double gloves, and pharmacy technicians’ training, exposure risks remain in the ancillary activities of pharmacy units (drug reception, medical-tray preparation, validation of the manufacturing records and preparations, drug stock control, etc.). The multiplicity of tasks performed, and a less-frequent changing of gloves, effectively increases exposure risk. Thus, the contamination observed in pharmacy units could not be related only to the compounding activity.

By including a wide range of healthcare professionals in this study, we demonstrated that workers, other than pharmacists, pharmacy technicians, and nurses, who did not handled ANDs, were also at risk of contamination. It is clearly apparent that auxiliary nurses, cleaners, and couriers can be exposed.

Concerning the factors that may affect workers exposure, our findings did not indicate a relation between pharmacy technicians’ or pharmacists’ exposure and the amount of annual drug-compounding. We also did not find a significant association between exposure and exposure groups. However, the unit turned out to be a statistically-significant parameter. Our data showed that oncology unit workers (nurses, auxiliary nurses, and cleaners) were more often contaminated than pharmacy-unit workers (pharmacists and pharmacy technicians). The nature of activities performed in pharmacy units was very different to those in oncology units. In oncology units, we often noticed a lack of knowledge of the ANDs exposure sources, resulting in nonexistent or inadequate protective measures. Regarding auxiliary nurses, exposure sources should be sought in patient-care operations, handling of excreta, and soiled bedding. The sources of exposure of cleaners are mainly to be found in skin contact with the various surfaces in the rooms to be cleaned. Regarding the couriers, they were probably contaminated when handling the containers or the infusion bags. We have shown that the containers used to transport the infusion bags were regularly contaminated. These containers were not cleaned frequently. In addition, we also observed that some couriers were in the habit of removing the infusion bags (generally without wearing gloves) from the containers and placing them on the benches of the nurses’ room (as a courteous service). Moreover, preventive measures were primarily aimed at nurses, who were considered to be at highest risk of exposure to ANDs. As a result, auxiliary nurses, cleaners, or couriers did not always have suitable personal protective equipment at their disposal, and their knowledge of ANDs issues was usually insufficient.

The use of environmental monitoring was valuable to assess the prevalence and levels of work-environment contamination. The wipe samples collected in pharmacies and oncology units indicated that contaminations by CP and 5FU were frequent for most surfaces. Work-environment contaminations have been reported on several occasions in the literature [14,25,26,27].

The most-contaminated surfaces were the outsides of gloves. These contaminations were then transferred to the surfaces of infusion bags and then to workbenches. Contamination of the surfaces of infusion bags could explain the contamination of pharmacists, who had to handle the infusion bags to validate the preparations.

Environmental monitoring enabled contaminations to be detected, less-known exposure sources to be identified, and worker contaminations to be sometimes explained. This was the case for the probable relation between infusion bags’ surface contamination and pharmacist and nurse exposures, between floors’ and patient-rooms’ surface contamination and cleaner exposures, or between contamination of closed containers to convey preparations and courier exposures. In this respect, environmental monitoring fosters workers’ awareness of potential exposure sources and of the usefulness of job procedures and protective equipment. Contamination of the work environment also raises the issue of unknown contamination. In some common work areas such as nurse rooms, wearing of PPE is in fact not expected when ANDs were not handled for administration. Thus, any person present in this environment is likely to come into contact with potentially-contaminated surfaces such as workbenches, telephones, or computer keyboards. It is therefore essential to keep the work environment free of chemical contamination.

## 5. Conclusions

This study documented occupational exposure of antineoplastic drugs in French medical centers. We demonstrated that although a wide range of healthcare workers were at risk of contamination, workers in oncology units were more frequently exposed than other HCWs. Therefore, risk-management measures must be implemented to reduce exposures and keep them at lowest-possible levels. First and foremost, all the occupational categories that may be directly or indirectly exposed should be identified. Exposing activities should be also identified. This is mandatory in order to provide regular and appropriate information on potential exposure sources and to adopt relevant preventive measures. Regular and adequate training should be implemented for pharmacy technicians and nurses. The training must include procedures in wearing and removing personal protective equipment. In addition, exposure assessment, including biological- and environmental-monitoring, is recommended to ensure the long-term efficiency of the prevention measures applied. Future research should include a larger number of ANDs to fully appreciate the extent of HCWs exposure. In addition, home-care nurses must be included given the development of home chemotherapy.

## Figures and Tables

**Table 1:** Main characteristics of medical centers and healthcare workers contamination by site.

	Medical Centers											
	1	2	3	4	5	6	7	8	9	10	11	12
**Central drug-compounding unit**	Yes	Yes	Yes	Yes	No	Yes	Yes	No	Yes	Yes	Yes	Yes
**Number of drug preparations/year**	20,000	14,000	13,500	20,000	7500	26,000	23,000	8500	3000	20,000	28,000	20,000
**Number of participants**	19	20	31	15	5	20	26	12	12	44	43	3
**Number of participants with positive samples (%)**	15 (79%)	1 (5%)	15 (48%)	8 (53%)	4 (80%)	15 (75%)	16 (61%)	9 (75%)	5 (42%)	23 (52%)	21 (49%)	0 (0%)
**Number of samples**	121	145	188	114	26	141	168	97	80	271	334	18
**Number of positive samples (%)**	37 (30%)	2 (1%)	26 (14%)	14 (12%)	6 (23%)	37 (26%)	29 (17%)	21 (22%)	6 (8%)	36 (13%)	36 (11%)	0 (0%)

**Table 2:** Ranges of ANDs concentrations (µg/L) in urine samples by hospital center.

Medical Centers	CP ^1^ LOQ ^5^: 0.05 µg/L	IF ^2^ LOQ: 0.1 µg/L	MTX ^3^ LOQ: 0.1 µg/L	FBAL ^4^ LOQ: 1 µg/L
**1**	0.06–0.99	0.1–0.25	-	1.00–22.7
**2**	-	-	0.50–0.88	-
**3**	0.05–0.42	-	0.15–1.42	1.01–6.03
**4**	0.05–0.75	0.16	-	1.08–11.0
**5**	-	-	-	1.23–7.86
**6**	0.05–0.86	0.10–0.18	0.10–0.15	1.00–7.62
**7**	0.05–0.29	0.10–0.44	0.10–1.73	1.00–14.3
**8**	0.05–0.75	0.11–0.11	--	1.00–7.83
**9**	0.11	0.20	-	1.09–14.6
**10**	0.05–0.50	0.10–0.15	0.13–3.17	1.10–8.83
**11**	0.05–0.81	0.10	0.15–2.25	1.00–24.5
**12**	-	-	-	-
**All**	0.05–0.99	0.10–0.44	0.10–3.17	1.00–24.5

^1^ CP = cyclophosphamide, ^2^ IF = ifosfamide, ^3^ MTX = methotrexate, ^4^ FBAL = α-fluoro-β-alanine, ^5^ LOQ = limit of quantification.

**Table 3:** Healthcare workers contamination by exposure group.

	Pharmacy Technicians	Pharmacists	Nurses	Auxiliary Nurses	Cleaners	Physicians	Others
**Number of participants**	56	16	104	48	14	4	8
**Number of participants with positive samples (%)**	25 (45%)	7 (44%)	57 (55%)	28 (58%)	7 (50%)	0 (0%)	8 (100%)
**Number of samples**	411	143	644	332	91	16	66
**Number of positive samples (%)**	45 (11%)	14 (10%)	112 (17%)	55 (17%)	14 (15%)	0 (0%)	10 (15%)

## Data Availability

The data presented in this study are available on request from the corresponding author. The data are not publicly available due to company privacy.
